# Dr. Ernest Byron Smith

**Published:** 1913-02

**Authors:** 


Dr. Ernest Byron Smith, Cleveland Homoeopathic Medical College, 1882, of Erie, Pa., died at the Buffalo General Hospital, December 11, 1912, from cancer, aged 52.

